# Genome-wide identification and expression analysis of the AUX/IAA gene family in turnip (*Brassica rapa* ssp. *rapa*)

**DOI:** 10.1186/s12870-023-04356-y

**Published:** 2023-06-27

**Authors:** Huanwen Xu, Yu Liu, Shengmei Zhang, Deju Shui, Zhewen Xia, Ji Sun

**Affiliations:** 1 College of Agriculture and Biotechnology, Wenzhou Vocational College of Science and Technology (Wenzhou Academy of Agricultural Sciences), Wenzhou, Zhejiang 325006 China; 2 Southern Zhejiang Key Laboratory of Crop Breeding, Wenzhou Vocational College of Science and Technology (Wenzhou Academy of Agricultural Sciences), Wenzhou, Zhejiang 325006 China; 3 Wenzhou Key Laboratory of Resource Plant Innovation and Utilization, Zhejiang Institute of Subtropical Crops, Zhejiang Academy of Agricultural Sciences, Wenzhou, Zhejiang 325005 China; 4 Wenzhou Lucheng District Agricultural Industry Institute, Wenzhou, Zhejiang 325000 China

**Keywords:** Genome-wide analysis, AUX/IAA gene family, Phylogenetic relationship, Expression analysis, *Brassica rapa* ssp. *rapa*

## Abstract

**Background:**

Auxin/indoleacetic acid (AUX/IAA) genes encoding short-lived proteins participate in AUX signaling transduction and play crucial roles in plant growth and development. Although the AUX/IAA gene family has been identified in many plants, a systematic analysis of AUX/IAA genes in *Brassica rapa* ssp. *rapa* has not yet been reported.

**Results:**

We performed a comprehensive genome-wide analysis and found 89 AUX/IAA genes in turnip based on the conserved AUX/IAA domain (pfam02309). Phylogenetic analysis of AUX/IAA genes from turnip, Arabidopsis, and cabbage revealed that these genes cluster into six subgroups (A1, A2, A3, A4, B1, and B2). The motif distribution was also conservative among the internal members of the clade. Enhanced yellow fluorescent protein (EYFP) signals of BrrIAA-EYFPs showed that BrrIAA members functioned as nucleoproteins. Moreover, transcriptional analysis revealed that the expression patterns of AUX/IAA genes in turnip were tissue-dependent. Because orthologs have similar biological functions and interaction networks in plant growth and development, *BrrIAA66* in turnip possibly played a role in embryo axis formation, vascular development, lateral root formation, and floral organ development by interacting with BrrARF19 and BrrTIR1.

**Conclusion:**

These results provide a theoretical basis for further investigation of *BrrAUX/IAA* genes and lay the foundation for functional analysis of *BrrIAA66* in turnip.

**Supplementary Information:**

The online version contains supplementary material available at 10.1186/s12870-023-04356-y.

## Background

Auxin is the first class of phytohormones to be discovered, and they play a crucial role in an extraordinarily broad spectrum of biological mechanisms ranging from basic cellular processes (e.g., cell division and expansion) to macroscopic phenomena (e.g., hypocotyl elongation, lateral root development, and biotic stress responses) [1–4]. Indole-3-acetic acid (IAA), the predominant naturally occurring auxin form in higher plants, plays a major role in biosynthesis, metabolism, transport, location, signal transduction, and cross-talk with other hormones [5–11]. Auxin signal transduction is the transfer of a signal throughout an organism, especially across or through a cell. The auxin signal transduction pathway in plants is not singular, and it involves several stages, including signal recognition, expression of downstream auxin-related genes, and physiological responses in plants. Downstream auxin-related genes, termed early/primary auxin response genes, include AUX/IAA family genes, auxin response factor (ARF) family genes, small auxin upregulated RNA [12], aminocyclopropane-1-carboxylic acid synthase [13], glutathione-S-transferase (GH2/4-like), and auxin-responsive Gretchen Hagen 3 (GH3) family genes, among others [14, 15].

Most AUX/IAA genes are rapidly and specifically induced by the exogenous plant hormone auxin, except for *AtIAA28* [16]. AUX/IAA proteins also function as transcriptional repressors and mediate AUX signaling by interacting with TIR1 receptors [17, 18]. Canonical AUX/IAA proteins usually share four conserved domains (I, II, III, and IV). Domain I at the N-terminus is an active, portable, and dominant repression domain, which is essential for interaction with TOPLESS co-repressors [19]. Domain II confers instability on AUX/IAA proteins by interacting with a degron sequence and TIR1/AFB protein [20, 21]. Domains I and II contribute to the AUX/IAA degradation rate, which is essential for plant growth and development [22, 23]. However, atypical AUX/IAA proteins lack domain II, which mediates canonical AUX signaling by the TIR1-dependent pathway [24–26]. Domains III and IV are responsible for homo- and hetero-dimerization of AUX/IAA or ARF proteins [27, 28].

Large-scale studies in recent years have revealed that AUX/IAA genes play a crucial role in the growth and development of organs such as the hypocotyl, stem, inflorescence, roots, and leaves, as well as in apical dominance. For instance, the overexpression of *AtIAA1* with a domain II mutation impairs cell elongation and division in the hypocotyl, stem, inflorescence, and leaves [29]. *AtIAA6*, *AtIAA9*, and/or *AtIAA17* form specific sensing complexes with *TIR1* and/or *AFB2* to modulate jasmonic acid (JA) homeostasis and consequent adventitious root initiation [30]. The SOR1-OsIAA26 (atypical gene) module acts downstream of OsTIR1/AFB2-AUX-OsIAA9 (canonical gene), signaling to modulate root-specific ethylene responses in rice [31]. The gain-of-function *iaa18-1* mutation in Arabidopsis increases the stability of AUX/IAA protein IAA18 and causes aberrant cotyledon placement in embryos [32].

Turnip (*Brassica rapa* ssp. *rapa*, 2*n* = 2*x* = 20), one of the three diploid Brassicaceae subspecies of family Cruciferae, is an important leaf and root vegetable crop for human consumption and animal fodder in East Asia including China. It has been cultivated in Zhejiang Province, China, and is locally called “Pancai”. Thus, genome-wide identification and characterization of the AUX/IAA gene family have been performed in various plant species, including *Arabidopsis thaliana* [33], *Solanum lycopersicum* [34], *Cucumis sativus* [35], *B. rapa* ssp. *pekinensis* [36], *S. tuberosum* [37], *B. napus* [38], and *Raphanus sativus* [39]. However, no genome-wide characterization of this gene family is available for turnip, resulting into the molecular mechanisms of AUX/IAA genes involved in turnip development remain unclear. Moreover, Huang et al. [40] reported that AUX plays a role in turnip hypocotyl-tuber formation in the early stages. Thus, studying the role of AUX/IAA genes in turnip can provide a better understanding of the functional attributes of this gene family in turnip and stimulate studies in related organisms.

In this study, 89 AUX/IAA members were identified in turnip. The phylogenetic relationships of AUX/IAA genes in Arabidopsis and *Brassica* were analyzed. Multiple amino acid alignment, conserved motif analysis, and subcellular localization analysis of AUX/IAA genes in turnip were performed. Furthermore, the expression patterns of AUX/IAA genes in different tissues were investigated. Moreover, the protein–protein interaction network of BrrIAA66 was predicted. These results will provide useful information for studies of turnip and other crops to unravel the functional involvement of the AUX/IAA gene family in diverse growth and development processes.

## Results

### Identification and analysis of AUX/IAA genes in turnip

To identify AUX/IAA family genes in turnip, AUX/IAA protein sequences of *Arabidopsis* and cabbage were downloaded and used as query sequences to search the turnip database and perform hidden Markov model construction and BLAST searches. Subsequently, the AUX/IAA family genes were verified after confirming the presence of the conserved AUX/IAA domain (pfam02309). Finally, 89 AUX/IAA genes were identified in turnip, which were designated as *BrrIAA1*–*BrrIAA89* (Additional file 1-S1).

AUX/IAA gene ID and physicochemical properties, including amino acid number, molecular weight, isoelectric point, instability index, aliphatic index, and grand average of hydropathy (GRAVY) value of the 89 AUX/IAA proteins were determined and are listed in Additional file 1-S1. Their amino acid sequence length ranged from 113 (*BrrIAA89*) to 1186 (*BrrIAA3*), and their isoelectric point ranged from 4.67 (*BrrIAA35*) to 9.44 (*BrrIAA64*). Their GRAVY value was negative, indicating that all 89 AUX/IAA proteins in turnip were hydrophilic. Based on Gene Ontology (GO) analysis, the 89 AUX/IAA genes were enriched in cell, organelle, and cell part of cellular component, and metabolic process, cellular process, signaling, response to stimulus, and biological regulation of biological process (Additional file 1-S1).

### Phylogenetic relationships of AUX/IAA genes in Arabidopsis and Brassica

**Fig. 1:**
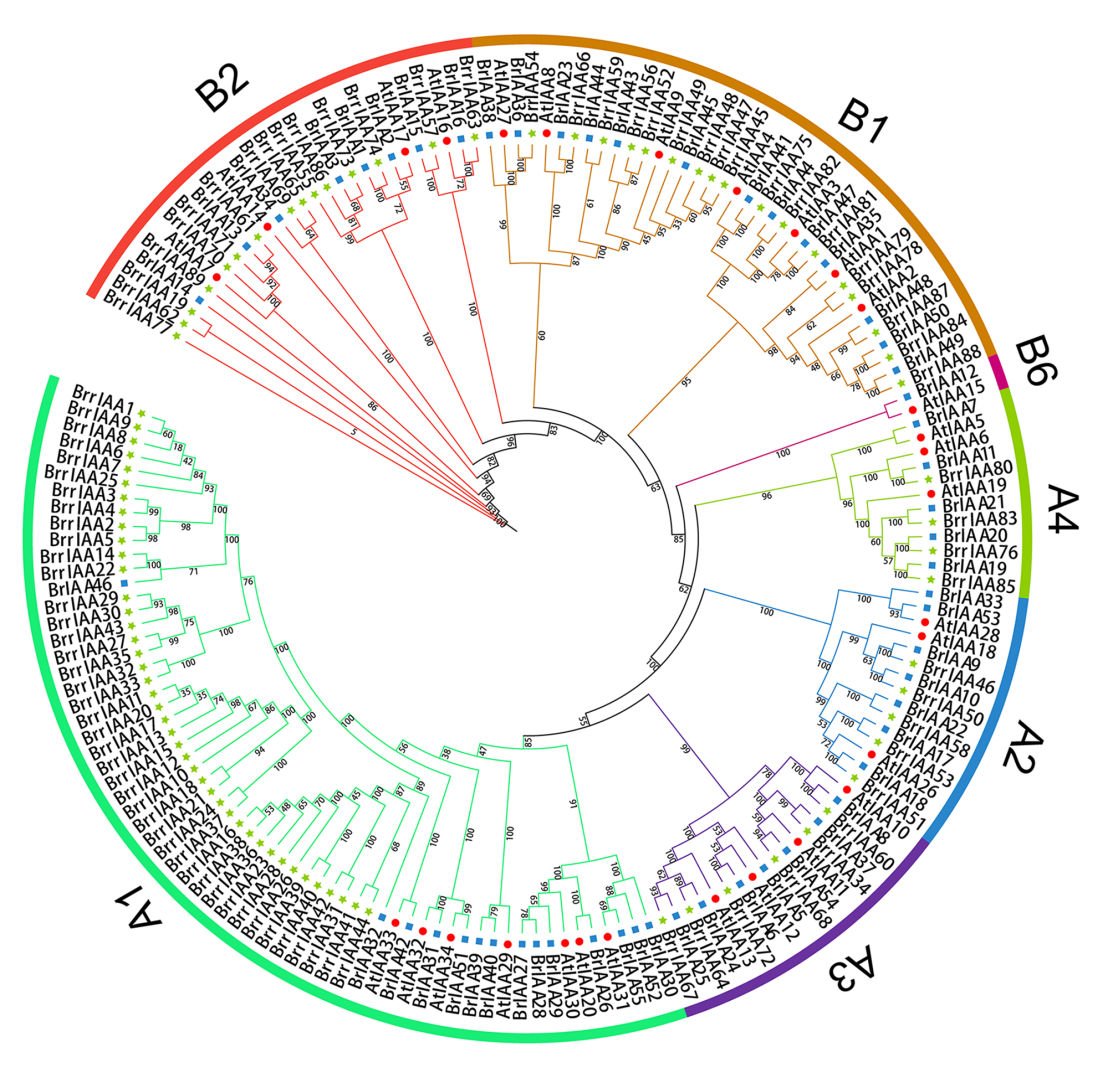
Phylogenetic relationships of AUX/IAA genes in *Arabidopsis thaliana*, *Brassica rapa* ssp. *pekinensis*, and *B. rapa* ssp. *rapa*. The neighbor-joining phylogenetic tree was generated using MEGA 7.0 based on the alignment of full-length amino acid sequences of 29 *(A) thaliana*, 55 *(B) rapa* ssp. *pekinensis*, and 89 *B. rapa* ssp. *rapa* AUX/IAA proteins. Seven subgroups were displayed by colored arcs. Subgroups A1, A2, A3, A4, B1, B2, and B6 were indicted by light green, blue, purple, green, brown, red, and rose red, respectively. The 29 orthologous sets and two *Brassica* specific sets are indicated by colored signs. AUX/IAA genes from *(A) thaliana*, *(B) rapa* ssp. *pekinensis*, and *B. rapa* ssp. *rapa* were indicated by red circles, blue squares, and green stars, respectively

To examine the phylogenetic relationships of the AUX/IAA gene family members, a neighbor-joining (NJ) phylogenetic tree was constructed using full-length amino acid sequences of 29 *AtAUX/IAA* genes from *Arabidopsis*, 55 *BrAUX/IAA* genes from cabbage, and 89 *BrrAUX/IAA* genes from turnip (Fig. 1). The phylogenetic tree was divided into groups A and B, which were further divided into four (A1, A2, A3, and A4) and three (B1, B2, and B6) subgroups, respectively. We found that 29 *AtAUX/IAA* genes from *Arabidopsis* and 55 *BrAUX/IAA* genes from cabbage were distributed in all seven subgroups, while 89 *BrrAUX/IAA* genes from turnip were clustered into six subgroups (except for subgroup B6). Group A contained 107 AUX/IAA genes, including 63, 15, 17, and 12 in subgroups A1, A2, A3, and A4, respectively. Moreover, subgroup A1 had a higher number of *AUX/IAA* genes from turnip than those of the other subgroups. Group B included 66 *AUX/IAA* genes, with 37, 27, and 2 in subgroups B1, B2, and B6, respectively. Notably, subgroup B6 only contained genes *BrIAA12* and *AtIAA15* from *Arabidopsis* and cabbage, respectively.

### Multiple amino acid alignment and motif distribution of AUX/IAA proteins in turnip

To clarify the sequence characteristics of AUX/IAA genes in turnip, multiple amino acid alignment of turnip AUX/IAA proteins was performed (Additional file 2). A total of 34 (38.20%) AUX/IAA proteins contained four canonical domains, 8 (8.99%) contained three domains (five with I, II, and III; three with II, III, and IV), 5 contained two domains (two with I and III; three with III and IV), and the remaining 42 (49.44%) only contained domain III. Notably, the proteins in the same subgroup have similar domain distribution. Almost all AUX/IAA proteins in subgroups A2, A3, A4, B1, and B2 were canonical proteins, whereas all AUX/IAA proteins in subgroup A1 were atypical proteins.

**Fig. 2:**
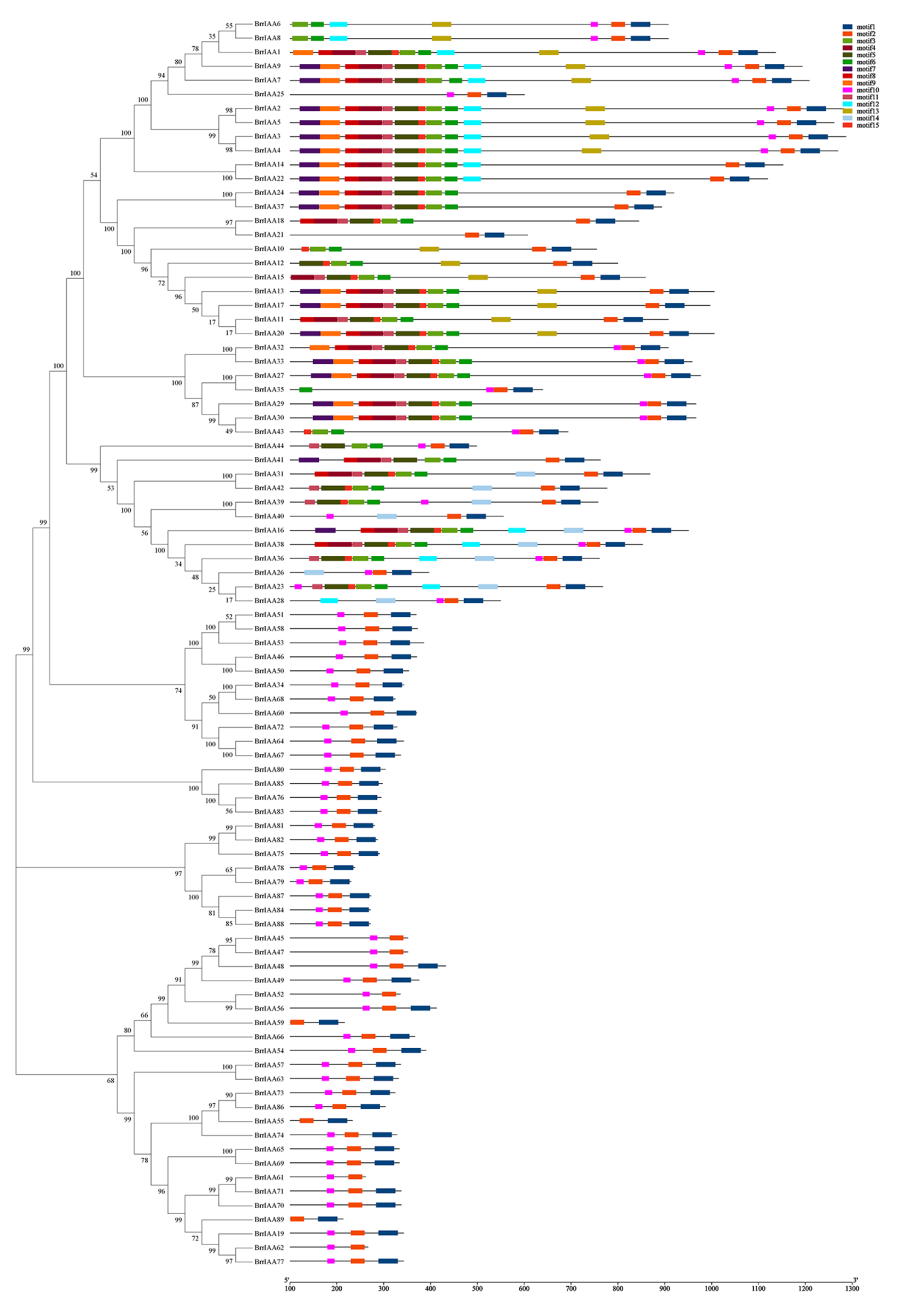
Conserved motif distribution of the *B. rapa* ssp. *rapa* AUX/IAA gene family. Motifs of AUX/IAA proteins were investigated using the MEME web server. The amino acid sequences of 15 motifs are shown in Additional File 3

To elucidate the similarity and diversity of AUX/IAA proteins, the possible conserved motifs were analyzed using MEME software. In total, 15 conserved motifs were detected, namely motifs 1–15 (Fig. 2; Additional file 3). All turnip AUX/IAA proteins included motif 2, which was included by domain III. The number of motifs contained in AUX/IAA proteins ranged from 2 to 14. Moreover, all AUX/IAA proteins in subgroups A2, A3, and A4 contained motifs 1, 2, and 10. In addition, the motif distribution was relatively conservative among the internal members of the clade. For instance, BrrIAA6 and BrrIAA8 harbored motifs 1, 2, 3, 6, 10, 12, and 13; BrrIAA2 and BrrIAA5 contained motifs 1, 2, 3, 4, 5, 6, 7, 8, 9, 10, 11, 12, 13, and 15; and BrrIAA51 and BrrIAA58 included motifs 1, 2, and 10.

### Synteny and Ka/Ks analysis

To explore collinearity of the 89 AUX/IAA genes in turnip, sequence similarity was analyzed using BLAST. In total, 3876 AUX/IAA gene pairs in turnip showed segmental repetition (Additional file 1- S2). Moreover, KaKs Calculator was used to measure their Ka/Ks values to reveal the evolutionary direction. Ka/Ks values of 13 gene pairs (*BrrIAA3*-*BrrIAA4*, *BrrIAA45*-*BrrIAA48*, *BrrIAA47*-*BrrIAA48*, *BrrIAA45*-*BrrIAA49*, *BrrIAA47*-*BrrIAA49*, *BrrIAA48*-*BrrIAA49*, *BrrIAA52*-*BrrIAA56*, *BrrIAA79*-*BrrIAA84*, *BrrIAA52*-*BrrIAA72*, *BrrIAA52*-*BrrIAA65*, *BrrIAA52*-*BrrIAA69*, *BrrIAA45*-*BrrIAA81*, and *BrrIAA47*-BrrIAA81) were higher than 1. Ka/Ks values of 3863 gene pairs were lower than 1 (Additional file 1- S2).

### Representative BrrIAA proteins were localized to the nucleus

**Fig. 3:**
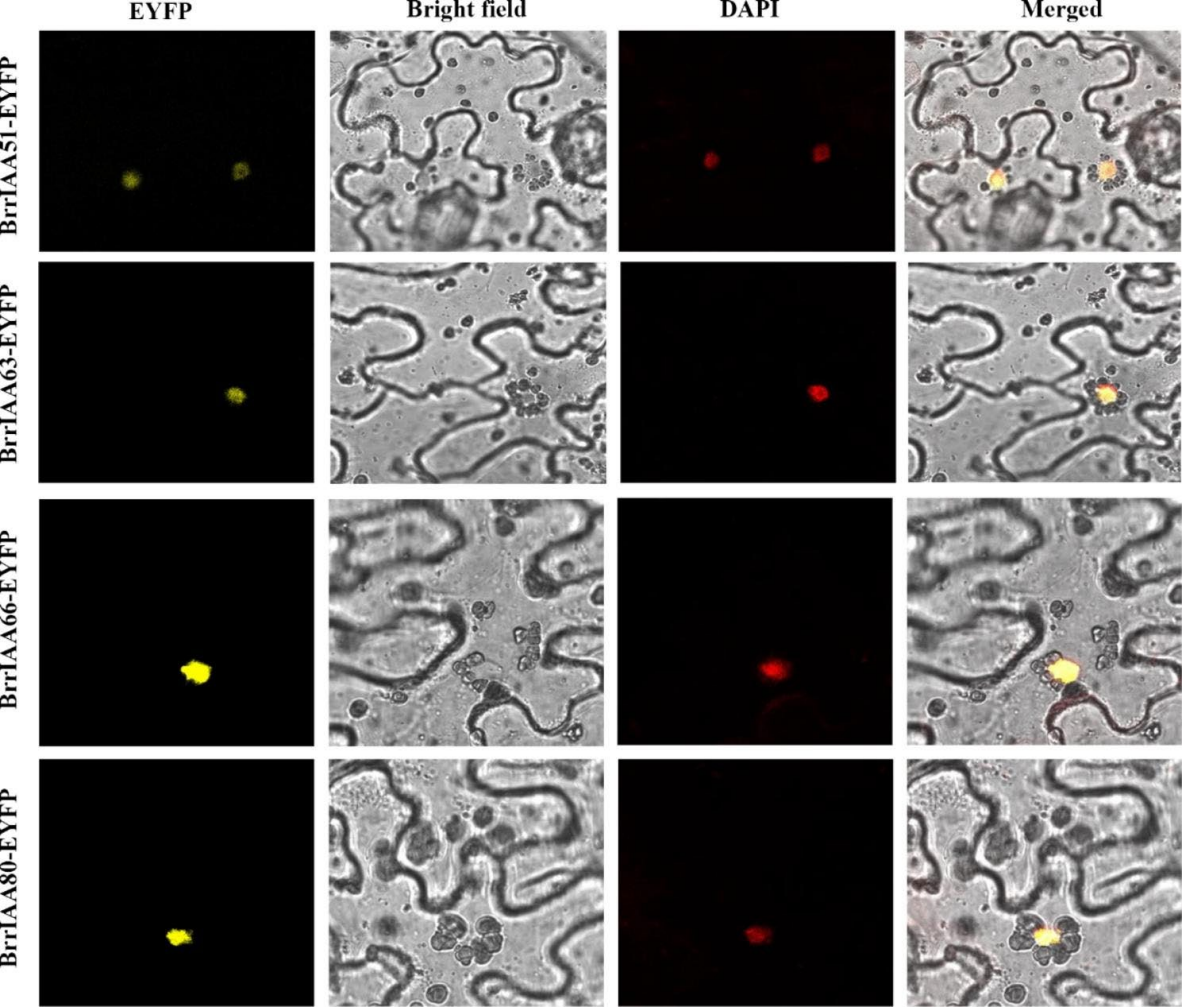
Subcellular localization of *35 S::BrrIAA-EYFP* in *Nicotiana benthamiana* leaves. BrrIAA-EYFP localized in the nucleus

Online protein prediction software was used to predict and confirm the subcellular localization of BrrIAA proteins. All BrrIAA proteins were predicted to localize to the nucleus (Additional file 1- S3). The enhanced yellow fluorescent protein (EYFP) gene was fused with BrrIAA51/63/66/80 as a reporter. By using laser confocal microscopy, EYFP signals of BrrIAA-EYFPs were detected in the nucleus (Fig. 3), suggesting that BrrIAA proteins functioned as nucleoproteins.

### Expression patterns of AUX/IAA genes in turnip and quantitative reverse transcription-polymerase chain reaction (qRT-PCR) validation

**Fig. 4:**
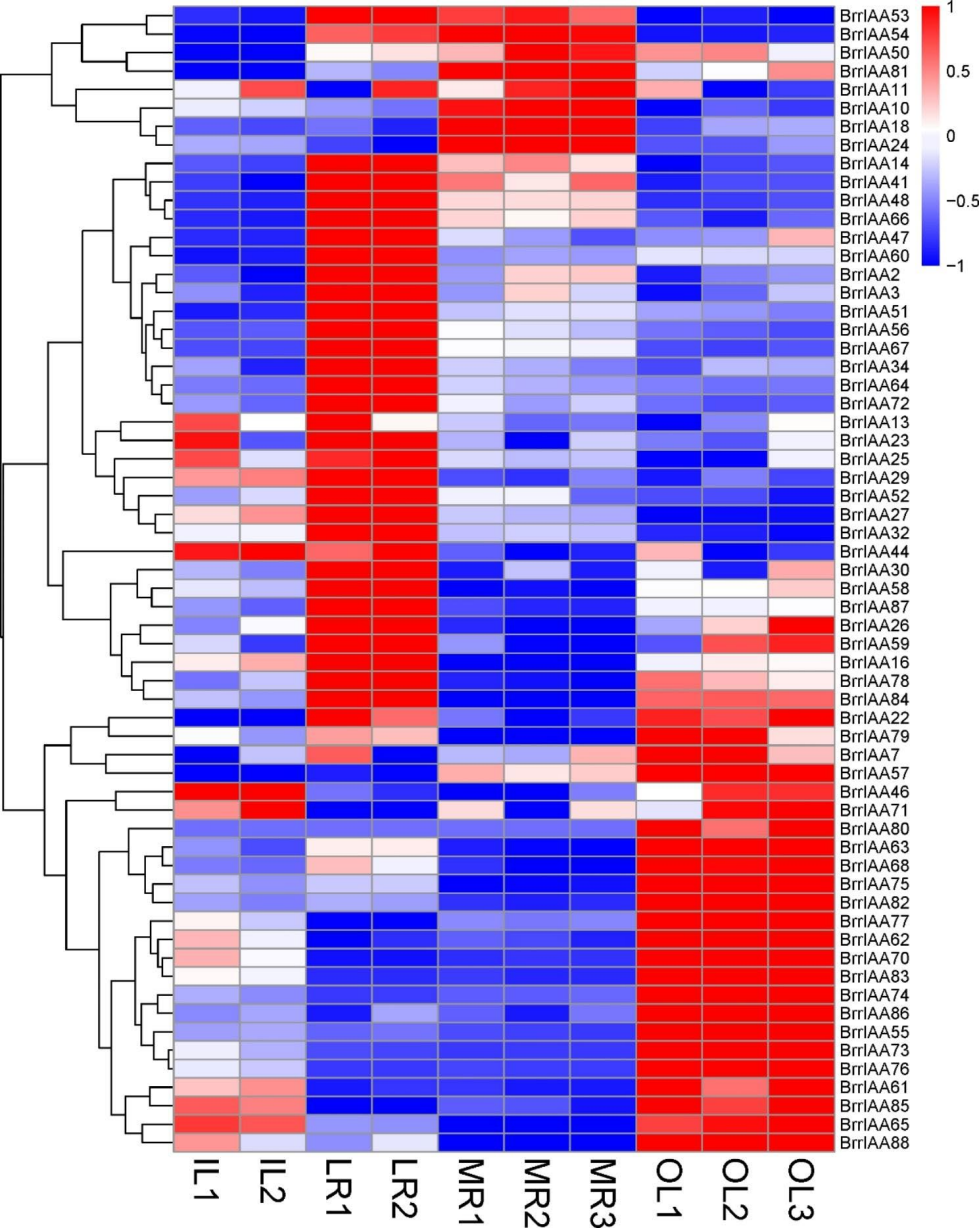
Heatmap of *B. rapa* ssp. *rapa* AUX/IAA family gene expression in 10 tissues (main and lateral roots, and outer and inner leaves). Expression values were calculated as fragments per kilobase of transcript per million mapped reads (FPKM). The scale represents normalized value of FPKM using the z-score method

The expression pattern of a gene is related to its function [41]. To gain insights into the putative functions of the 89 *AUX/IAA* genes in turnip development, we analyzed their expression patterns in four major tissues, including lateral roots, main root, and outer and inner leaves, based on RNA-sequencing (RNA-seq) data. The results indicated that the expression patterns of *BrrAUX/IAA* genes in different tissues were different. A total of 62 *BrrAUX/IAA* genes were expressed in the main and lateral roots, and outer and inner leaves, of which 37 genes had higher expression in roots than in leaves (Fig. 4; Additional file 1- S4 and S5). For instance, *BrrIAA10*, *BrrIAA18*, *BrrIAA24*, *BrrIAA50*, *BrrIAA54*, and *BrrIAA81* were expressed at high levels in the main root. *BrrIAA16*, *BrrIAA48*, *BrrIAA53*, *BrrIAA66*, *BrrIAA84*, and *BrrIAA87* were highly expressed in the lateral roots. A total of 25 genes were expressed at higher levels in leaves than in roots (Additional file 1- S6). For example, *BrrIAA63*, *BrrIAA70*, *BrrIAA73*, *BrrIAA74*, *BrrIAA75*, and *BrrIAA77* were mainly expressed in the outer leaves. *BrrIAA44*, *BrrIAA46*, *BrrIAA65*, and *BrrIAA71* had higher expression in the inner leaves than in the other tissues. However, low expression levels of 27 genes were observed in the four tissue types (Additional file 1- S7).

**Fig. 5:**
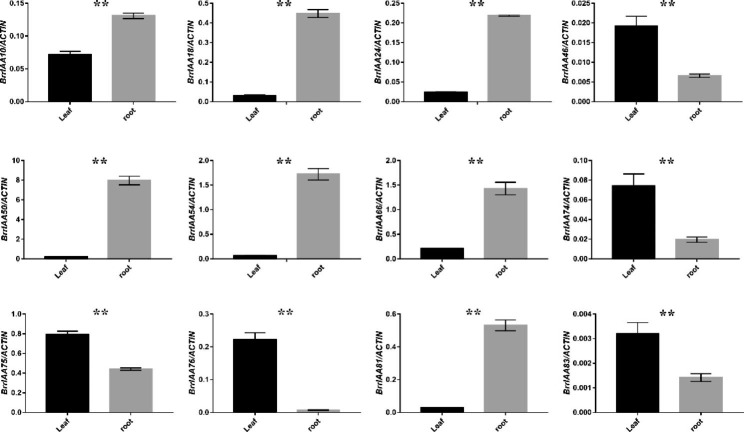
Verification of 12 differentially expressed genes by qRT-PCR. Values are shown as means ± SD

To validate the RNA-seq results, qRT-PCR was performed to examine the expression of 12 randomly selected differentially expressed genes (DEGs). Of these DEGs, *BrrIAA10*, *BrrIAA18*, *BrrIAA24*, *BrrIAA50*, *BrrIAA54*, *BrrIAA66*, and *BrrIAA81* had significantly higher expression in roots than in leaves; in contrast, the relative expression levels of *BrrIAA46*, *BrrIAA74*, *BrrIAA75*, *BrrIAA76*, and *BrrIAA83* were significantly higher in leaves than in roots (Fig. 5).

### Protein–protein interaction prediction and yeast two-hybrid assay validation

*BrrIAA66* was expressed at a higher level in roots than in leaves, suggesting that it played a role in root growth and development. Using multiple sequence alignment and phylogenetic analysis, *BrrIAA66* was identified as the ortholog of *AtIAA8* (*AT2G22670*) and *BrIAA23* (*Bra030219*). Functional characterization and interaction networks of BrIAA23 and AtIAA8 probably provide clues for further investigation into functional validation and regulatory pathways of BrrIAA66. Therefore, the protein–protein interaction network of BrIAA23 and AtIAA8 was analyzed using the default parameters of STRING.

**Fig. 6:**
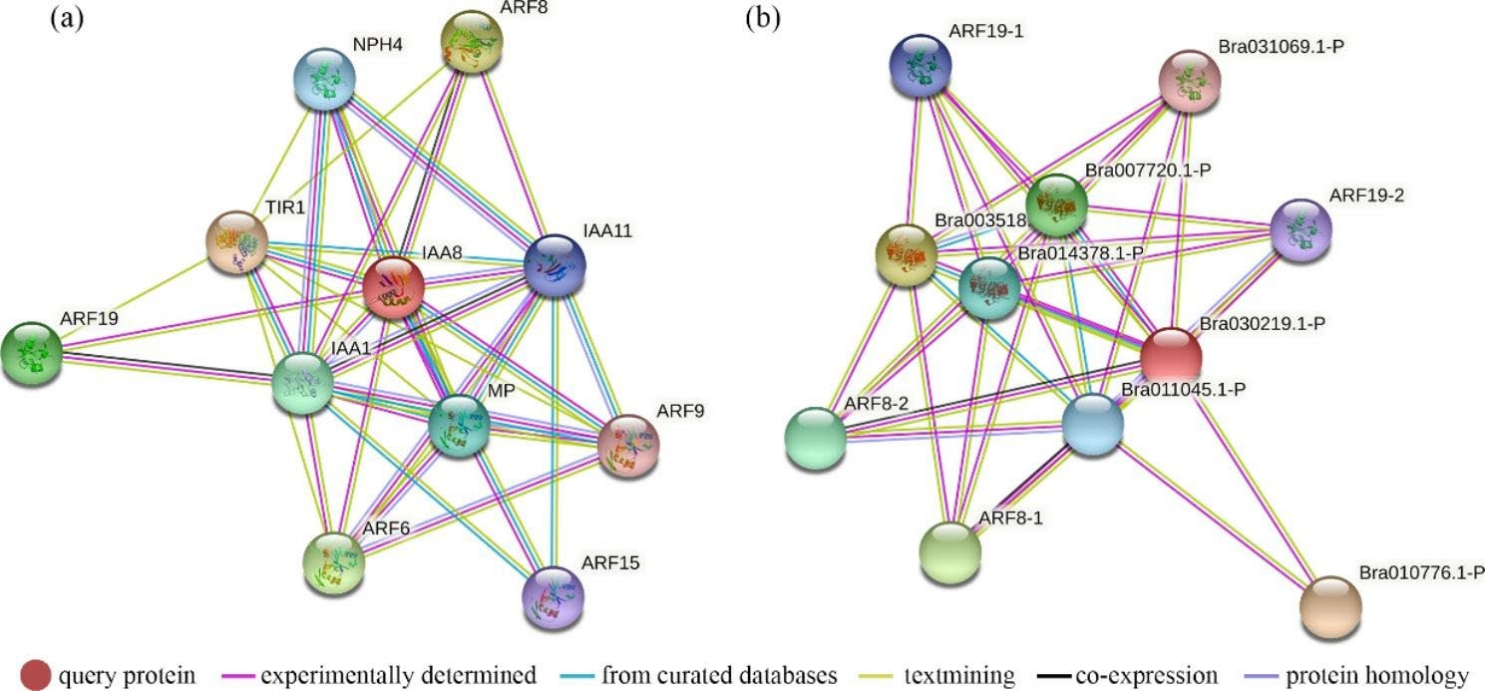
Predicted protein–protein interaction network of *AtIAA8* (*AT2G22670*) and *BrIAA23* (*Bra030219*). **a.** *AtIAA8* **b.** *BrIAA23*

**Fig. 7:**
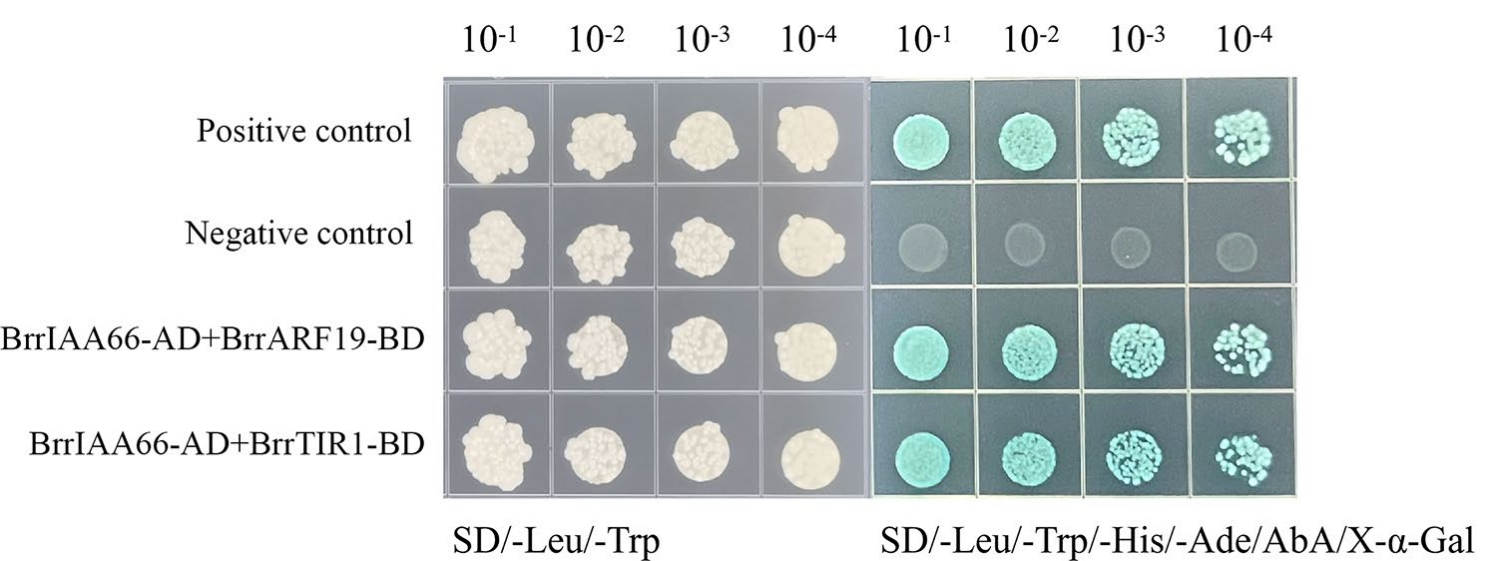
Yeast two-hybrid assays showing that BrrIAA16 interacts with BrrARF19 and BrrTIR1 to form heterodimers. Plasmids for negative controls were pGADT7-T and pGBKT7-Lam, and plasmids for positive controls were pGADT7-T and pGBKT7-53

AtIAA8 interacted with seven AtARFs (MP/ARF5, AtARF6, NPH4/ARF7, AtARF8, AtARF9, AtARF15, and AtARF19), two AtIAA proteins (AtIAA1 and AtIAA11), and TIR1 (Fig. 6a). BrIAA23 (Bra030219) interacted with four BrARFs (ARF8-1, ARF8-2, ARF19-1, and ARF19-2), Bra011045, Bra031069, Bra010776, Bra003518, Bra014378, and Bra007720 (Fig. 6b). Annotation of the preceding six genes was as follows: AUX-responsive protein IAA11, ethylene-responsive protein ERF17-like, AUX-responsive factor ARF6, transport inhibitor response 1, transport inhibitor response 1, and transport inhibitor response 1, respectively. In addition, yeast two-hybrid assay showed that BrrIAA66 formed heterodimers with BrrARF19 and BrrTIR1 (Fig. 7).

## Discussion

IAA was a phytohormone that regulates plant growth and development. AUX/IAA gene families, or early/primary auxin response genes, were rapidly and specifically induced by IAA. Hence, the characterization of AUX/IAA gene families has been useful for studying their functions in plants. In the present study, 89 AUX/IAA genes were identified in turnip. The number of identified AUX/IAA genes in turnip was higher than that in Arabidopsis (29) [42], cabbage (55) [36], potato (36) [37], peach (23) [43], cucumber (29) [35], tomato (26) [34], maize (31) [44], papaya (18) [45], and radish (65) [39], but lower than that in rapeseed (119) [38], despite their genome size. *B. rapa* had undergone various polyploidization events (γ, β, and α duplication events) and a whole-genome triplication (WGT) event, which has resulted in a unique diploidization process by genome fractionation and rearrangements [46–48]. Theoretically, *B. rapa* had roughly triple the number of genes as *A. thaliana*, which suggested that it has about 90 AUX/IAA genes, whereas *A. thaliana* had 29 AUX/IAA genes [49, 50]. In this study, the number of AUX/IAA genes in turnip was almost identical to this speculation, suggesting that the expansion of the AUX/IAA gene family in turnip was affected by segmental duplication resulting from *Brassica* WGT and allopolyploidy. Phylogenetic relationships based on chloroplast protein-coding sequences showed that turnip was most closely related to cabbage [51]. However, the number of AUX/IAA gene family members between turnip and cabbage was different because of alternative evolutionary histories during triplication.

Further evolutionary relationships of the AUX/IAA gene family members with Arabidopsis (29 AtAUX/IAA genes), cabbage (55 BrAUX/IAA genes), and turnip (89 BrrAUX/IAA genes) were examined (Fig. 1). The constructed phylogenetic tree contained two major groups, A and B, which were divided into seven subgroups (A1, A2, A3, A4, B1, B2, and B6). Major differences between cabbage and turnip were found in subgroup A1, which included 14 BrAUX/IAA genes and 42 BrrAUX/IAA genes (*BrrIAA1*–*BrrIAA44*, except for *BrrIAA19* and *BrrIAA34*), respectively. The higher load of AUX/IAA genes from turnip in subgroup A1 may played a vital role in the formation of enlarged roots. Further insights into phylogeny indicated 10 sister pairs from cross-species (*BrrIAA-BrIAA-AtIAA*), including one for B2, four for B1, one for A2, three for A3, and one for A4. Orthologs in the same clade had analogous biological functions in plant growth and development. For instance, *IAA3/SHY2* (*At1g04240*) played an important role in controlling root meristem size and root growth, indicating that *BrIAA47* and *BrrIAA81* may had similar roles in each species [52]. Notably, subgroup B6 only harbored two genes (*AtIAA15* and *BrIAA12*), which was in accordance with previous findings [36]. In multiple amino acid alignment (Additional file 2), 55 AUX/IAA proteins were found to lack single or multiple domains that may contributed to their functional divergence, whereas 48 AUX/IAA proteins did not contain domain I, suggesting that these proteins had lost the capacity to recruit TOPLESS co-repressors and did not contribute to classical AUX signal transduction. Dreher et al. [22] reported that *AtIAA20* lacking domain II cannot be rapidly degraded in the presence of basal or increased levels of AUX. In the present study, 47 AUX/IAA proteins lacking domain II were considered atypical proteins. Of these, 42 AUX/IAA proteins that were classified into subgroup A1 lack domains I, II, and IV. These proteins may interacted with additional unknown components and be degraded by a process in the AUX signal transduction cascade. Thus, these 42 AUX/IAA proteins from subgroup A1 probably had important roles during root enlargement in turnip, which was consistent with the results from the phylogenetic tree. Ka/Ks values of 13 gene pairs were higher than 1, revealing that these genes recently experienced tachytelic evolution. Ka/Ks values of 3863 gene pairs were lower than 1, suggesting that these genes experienced purity selection to reduce protein conformational and functional changes resulting from base nonsynonymous substitution.

Similarities and differences in AUX/IAA genes may resulted from their expression patterns produced by their promoter activity and/or molecular properties of their gene products [53]. Therefore, the expression patterns of the 89 AUX/IAA genes were analyzed in four major tissues. Distinct expression patterns of AUX/IAA genes among different tissues of turnip were observed, suggesting that the expression patterns of AUX/IAA genes in turnip were tissue-dependent (Fig. 4). For instance, *BrrIAA24*, *BrrIAA50*, *BrrIAA54*, and *BrrIAA81* had much higher expression in the main and lateral roots, while *BrrIAA46*, *BrrIAA57*, *BrrIAA65*, and *BrrIAA77* were expressed at higher levels in the outer leaves. Many studies showed that homologous genes may had functional similarities among different species. In our research, *BrrIAA54*, which was highly expressed in roots, may had a similar function to its homolog *AtIAA27* in Arabidopsis, which promoted lateral root development [54]. Similarly, *BrrIAA50*, whose homolog IAA18 exhibited high expression in melon roots, may be involved in regulating lateral root formation in melon [55]. In addition, these genes probably played important roles in hormone signal transduction pathways. For example, the gene function of *BrrIAA24* was likely similar to that of its homolog *IAA31* in Arabidopsis, which had high expression levels in the apical meristem of stems and roots and was believed to be involved in regulating the AUX signal transduction pathway [56]. In our research, *BrrIAA24* had much higher expression in the main and lateral roots. Root potentially had a high auxin accumulation in root tips to compare with shoot, what can be the reason of high expression of the *BrrIAA24* (Fig. 4) [57]. Moreover, these genes probably had additional roles in modulating biotic or abiotic stress signaling. For instance, *BrrIAA57*, whose homolog *IAA16* was expressed in roots and leaves of Arabidopsis, was involved in abscisic acid-regulated responses to biotic and abiotic stresses [58]. Besides, the expression patterns of AUX/IAA genes were also cell type-dependent. For instance, maize AUX/IAA gene, *RUM1*, expressed in pith cells around the xylem and involved in seminal and lateral root formation [59].

Orthologs usually have similar biological functions and interaction networks in plant growth and development. Thus, the biological function and regulatory pathway of the numerous AUX/IAA genes in Arabidopsis and cabbage were well studied mostly through mutations [32, 60–62], which probably provided a valuable framework for further functional and regulatory pathway prediction of *AUX/IAA* genes in turnip. For example, the characterization of gain-of-function mutations revealed that *AtIAA8* (*At2g22670*) was involved in root gravitropism, stem elongation, hair development, and apical dominance [63], indicating that *BrrIAA66*, the orthologs of *AtIAA8* and *BrIAA23*, may had similar functions in plant development. The interaction networks of AtIAA8 and BrIAA23 were analyzed using STRING. AtIAA8 and BrIAA23 interacted with ARF, IAA, and TIR1 proteins, which were relatively conserved, except for ERF17 (Fig. 6). Moreover, the result of yeast two-hybrid assay revealed that BrrIAA66 in turnip functioned by interacting with BrrARF19 and BrrTIR1 proteins. MP/ARF5 mediated embryo axis formation and vascular development [64]. NPH4/ARF7 was involved in differential growth responses of the hypocotyl and in lateral root formation [65]. *AtIAA8* was involved in lateral root formation by interacting with *TIR1* AUX receptors and *ARF* transcription factors [66, 67]. In addition, *AtIAA8* played a role in floral organ development by changing JA levels, probably by interacting with the ARF6/8 protein [63]. Therefore, *BrrIAA66* in turnip possibly played a role in embryo axis formation, vascular development, lateral root formation, and floral organ development.

## Conclusion

In this study, AUX/IAA gene family members were systematically identified in *Brassica rapa ssp. rapa*. The phylogenetic analysis showed that the AUX/IAA genes in turnip were divided into six subgroups. The motif distribution analysis indicated that the motif distribution of AUX/IAA proteins was conservative among the internal members of the clade. The expression patterns of AUX/IAA genes in turnip were tissue-dependent and probably functioned in the development of leaves and roots. Based on similar biological functions and interaction networks in plant growth and development among orthologs, *BrrIAA66* may play a role in embryo axis formation, vascular development, lateral root formation, and floral organ development by interacting with BrrARF19 and BrrTIR1 in turnip.

## Materials and methods

### Plant materials and growth conditions

Turnip plants from the elite line ‘dapan21hao’ were grown in a controlled chamber at 25 °C, 60% humidity, and photoperiodic lighting (16 h light/8 h dark). Leaf and root tissues were collected from at least three plants per biological replicate at 50 days after sowing, which were used for RNA extraction.

### Genome-wide identification of AUX/IAA proteins in turnip

AUX/IAA protein sequences of Arabidopsis and cabbage were downloaded from the *Arabidopsis* Information Resource (TAIR) and Brassicaceae Database (BRAD), respectively. Raw sequencing data of turnip was retrieved from the National Center for Biotechnology Information (NCBI) database using accession number PRJNA616845. Subsequently, candidate AUX/IAA members in turnip were identified by comparing HMMER 3.0 and BLASTP [68] with an e-value lower than 1e-20 as the threshold. By querying the PfamScan and Pfam A databases, all putative sequences were verified for the presence of the conserved AUX/IAA domain (pfam02309) [69]. Finally, the resulting sequences were defined as AUX/IAA members in turnip. The physicochemical properties of AUX/IAA proteins, including amino acid number, molecular weight, isoelectric point, instability index, aliphatic index, and GRAVY value were calculated using the ProtParam tool in ExPASy. GO annotation of the 89 AUX/IAA genes was also performed. MCScanX software and KaKs Calculator were used to draw the collinearity graph and obtain Ka/Ks values, respectively.

### Phylogenetic tree construction and conserved motif analysis

All AUX/IAA protein sequences of Arabidopsis, cabbage, and turnip were aligned using MAFFT with the default parameters [70]. The NJ phylogenetic tree was constructed using MEGA 7.0 software [71]. The parameters were set as follows: *p*-distance model of amino acid substitution type, partial deletion (gaps/missing data treatment), 50% cutoff, and a bootstrap test with 1000 replicates.

To better understand the similarity and diversity of AUX/IAA proteins, all amino acid sequences of AUX/IAA proteins were analyzed for conserved motifs using the online MEME suite [72]. The following parameters were applied: optimum motif widths of 6–50 residues and a maximum of 15 motifs. The schematic diagram of the amino acid motifs for each AUX/IAA protein was drawn according to a previous report [38].

### Subcellular localization of BrrIAA-EYFP fusion proteins

The coding sequence of BrrIAA51/63/66/80 was inserted into the pBI121-EYFP vector downstream of the CaMV 35 S promoter at the multiple cloning site. The constructs were transformed into Agrobacterium tumefaciens strain GV3101. *Nicotiana benthamiana* plants were grown at 22 °C under 16 h light/8 h dark conditions. Four-week-old plants were used for infiltration. Two days before infiltration, 2 mL of Agrobacterium strain culture was added to Luria–Bertani medium [10 mM morpholineethanesulfonic acid (MES), 40 μM acetosyringone (AS), 100 μM Kanamycin, and 50 μM rifampicin] at a 1:100 ratio and grown at 28 °C incubator with 180 rpm/min until it turned orange. Subsequently, cells were harvested by centrifugation at 6000 rpm for 10 min. Cell pellets were resuspended in infiltration medium (10 mM magnesium dichloride, 10 mM MES, and 150 µM AS) with OD_600_ adjusted to 1. Resuspended cell cultures were kept at room temperature for 3 h. Bacterial suspensions were infiltrated into the abaxial surface of fully expanded young leaves using a needleless syringe. After infiltration, plants were grown in darkness for 12 h and then in 16 h light/8 h dark for 48–72 h at room temperature. Subsequently, subcellular locations of proteins were monitored under a confocal laser scanning microscope (LSM 900; Carl Zeiss, Heidenheim, Germany) equipped with the filter units for YFP (Ex. 480 nm; Em. 527 nm). To locate the fluorescent proteins in nuclei, the *N. benthamiana* leaves were infiltrated with PBS containing 4′,6′-diamidino-2-phenylindole (DAPI) and cultured in dark culture about 2 minutes. The location of nuclei was monitored under 500 nm.

### Expression pattern analysis

RNA-seq data of four tissue types (lateral root, main root, outer leaf, and inner leaf) in turnip were downloaded from the NCBI database using SRA searches (SRX9799247 and SRX9799248 for inner leaf; SRX9799249, SRX9799250, and SRX9799251 for outer leaf; SRX9799252, SRX9799253, and SRX9799254 for the main root; SRX9799255 and SRX9799256 for lateral roots). Fragments per kilobase of transcript per million mapped reads (FPKM) were calculated using Cufflinks with default parameters to estimate gene expression levels. The heatmap was constructed using the pheatmap and ggplots R packages. Moreover, each value was transformed as a z-score to reduce the differences between the data.

### RNA extraction and qRT-PCR analysis

After sampling, leaf and root tissues were flash-frozen using liquid nitrogen and stored at − 80 °C. Total RNA was extracted using the standard TRIzol RNA isolation protocol (Invitrogen, Carlsbad, CA, USA). For each sample, approximately 1 µg RNA was used for reverse transcription with HiScript II Q RT SuperMix for qPCR (+ gDNA wiper) (Vazyme Biotech Co., Ltd., Nanjing, China) according to the manufacturer’s instructions. qRT-PCR was performed using AceQ® qPCR SYBR Green Master Mix (Vazyme Biotech) on a CFX96 real-time system (Bio-Rad Laboratories, Hercules, CA, USA). The PCR procedure included one cycle at 95 °C for 5 min and 40 cycles of 95 °C for 15 s, 60 °C for 20 s, and 72 °C for 20 s, followed by a melting curve program. The turnip *BrrACTIN* gene was used as endogenous control [73]. The specific primer pairs used for amplification are listed in Additional file 1- S8. Relative expression levels of genes were quantified using the 2^−ΔCt^ method with *BrrACTIN* as endogenous control [74]. All qRT-PCR assays were performed in three independent biological replicates.

### Protein–protein interaction prediction

The protein–protein interaction relationships between AtIAA8 and BrIAA23 were predicted using the STRING website.

### Yeast two-hybrid assay

Full-length *BrrIAA66* was inserted into the prey vector pGADT7. Full-length *BrrARF19* and *BrrTIR1* were cloned into the bait vector pGBKT7. The two vectors were co-transformed into yeast Y2HGold (Weidi Biotechnology, Shanghai, China) and cultured on double dropout medium lacking Trp and Leu (Coolaber, Beijing, China) at 30 °C for 2–3 days. Yeast colonies were selected on quadruple dropout medium lacking Leu, Trp, His, and Ade (Coolaber) with 100 ng/mL aureobasidin A to identify those with positive protein–protein interactions. Empty vectors were used as controls.

## Electronic supplementary material

Below is the link to the electronic supplementary material.

Supplementary Material 1

Supplementary Material 2

Supplementary Material 3

## Data Availability

Raw sequence data of *Brassica rapa* ssp. *rapa* was downloaded from the NCBI database using accession number PRJNA616845 (https://www.ncbi.nlm.nih.gov/bioproject/616845). RNA-seq data were derived from the NCBI database using accession number PRJNA690160 (https://www.ncbi.nlm.nih.gov/bioproject/PRJNA690160/). All datasets generated in this study are included in the published article/Additional Files. Websites used for analyses in this study are as follows: TAIR (https://www.arabidopsis.org/), BRAD (http://brassicadb.cn/), HMMER 3.0 (http://hmmer.janelia.org/), PfamScan and Pfam A (http://pfam.xfam.org/), ExPASy (http://web.expasy.org/protparam/), R (https://cran.r-project.org), STRING (http://string-db.org/), KaKs Calculator (http://code.google.com/p/kaks-calculator/wiki/kaks_Calculator). Access to these databases or websites is open. No new sequence data was generated in this study.
