# Supplementary material for: Genome-wide identification and expression analysis of the AUX/IAA gene family in turnip (Brassica rapa ssp. rapa)
Source: BMC Plant Biol. 2023 Jun 27;23:342. doi: 10.1186/s12870-023-04356-y (PMC10294438; doi:10.1186/s12870-023-04356-y)
Supplement: Supplementary file 3 — Supplementary Material 3 [file 12870_2023_4356_MOESM3_ESM.pdf]

Motif 1

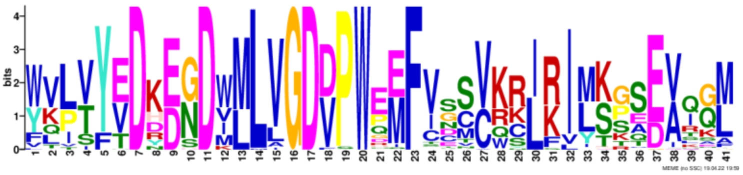

Motif 2

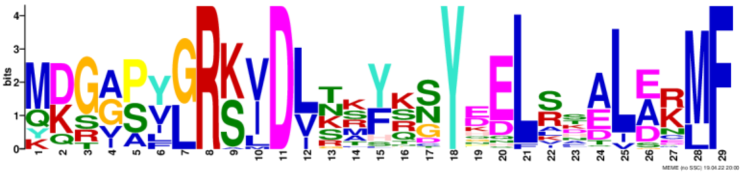

Motif 3

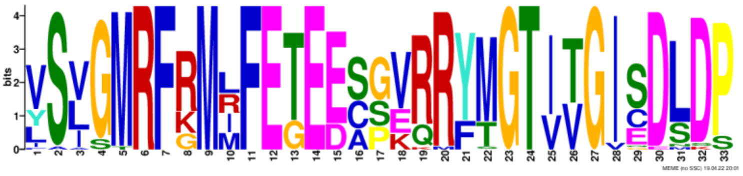

Motif 4

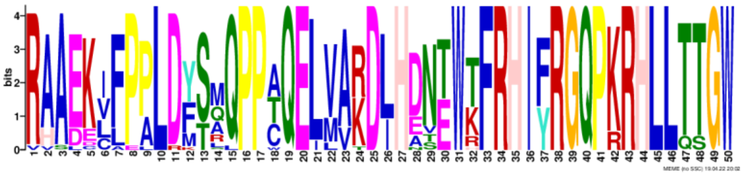

Motif 5

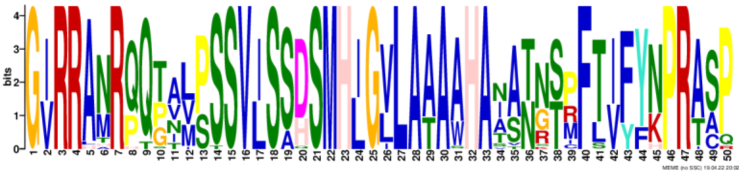

Motif 6

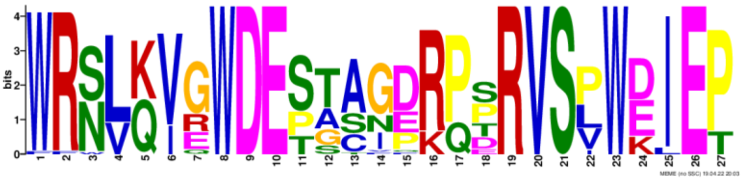

Motif 7

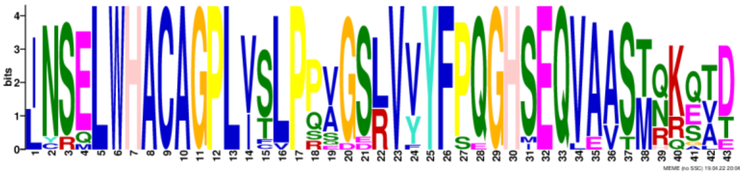

Motif 8

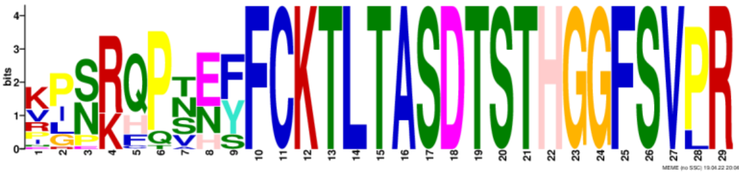

Motif 9

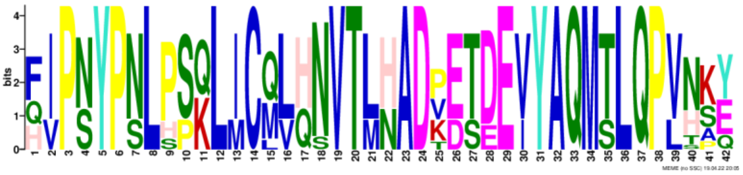

Motif 10

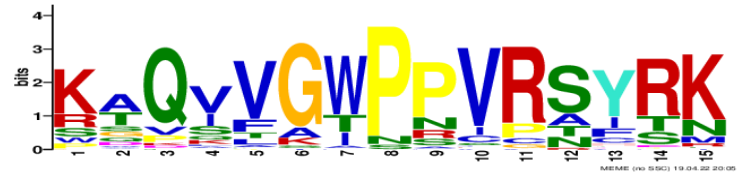

Motif 11

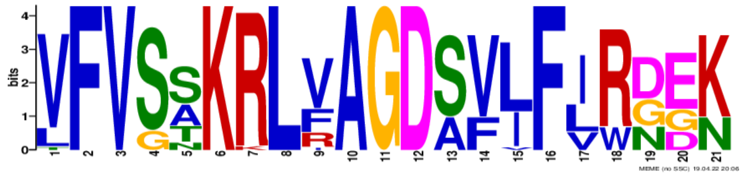

Motif 12

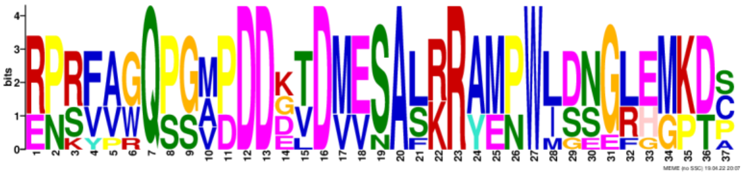

Motif 13

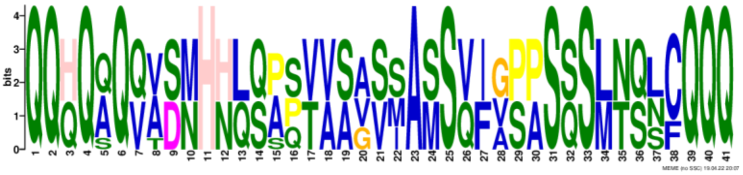

Motif 14

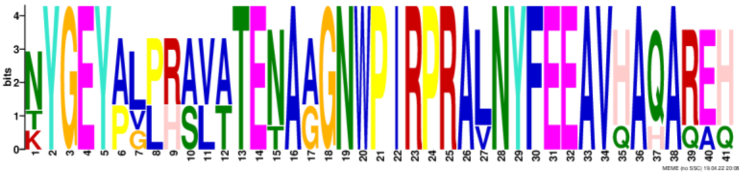

Motif 15

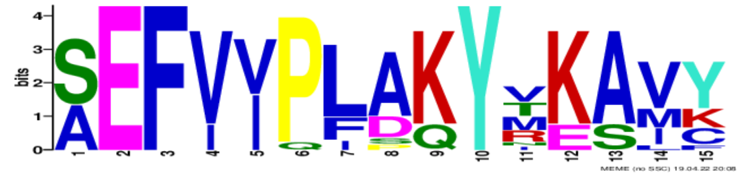

Addition file 3 The amino acid sequence of 15 conserved motifs
