# Supplementary material for: Genome-wide identification and expression analysis of the AUX/IAA gene family in turnip (Brassica rapa ssp. rapa)
Source: BMC Plant Biol. 2023 Jun 27;23:342. doi: 10.1186/s12870-023-04356-y (PMC10294438; doi:10.1186/s12870-023-04356-y)
Supplement: Supplementary file 2 — Supplementary Material 2 [file 12870_2023_4356_MOESM2_ESM.pdf]

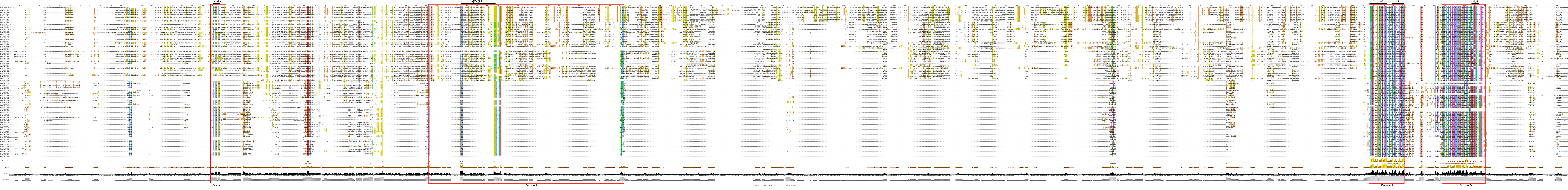
LxLxLx
GWXPP
β
α1
α2
NLS
Domain I
Domain II
Domain III
Domain IV
